# Explainable artificial intelligence in anesthesiology prediction models: bridging the gap from black-box algorithms to clinical decision support

**DOI:** 10.3389/fmed.2026.1928394

**Published:** 2026-08-20

**Authors:** Yan Wang, Zuoyan Song, Hang Wang

**Affiliations:** Department of Anesthesiology, Qingdao Municipal Hospital, Qingdao, China

**Keywords:** anesthesiology, clinical decision support, explainable artificial intelligence, machine learning, perioperative prediction, SHAP

## Abstract

Machine learning prediction models consistently outperform conventional clinical scoring tools across perioperative outcomes, yet remain largely unadopted at the bedside. This narrative review examines explainable artificial intelligence (XAI) as a bridge between algorithmic performance and clinical translation, drawing on a targeted search of PubMed and Embase (January 2018 to March 2026). Five prediction domains are synthesized: perioperative hypotension, postoperative complications, difficult airway management, depth-of-anesthesia monitoring, and regional anesthesia and pain management. SHAP (SHapley Additive exPlanations) has emerged as the dominant XAI method, identifying modifiable intraoperative targets. Attention mechanisms and GradCAM provide complementary interpretability for time-series and imaging-based models. Critical gaps persist: most delirium prediction models include no XAI analysis; no standardized evaluation framework exists, and no prospective trial has demonstrated that XAI-enhanced recommendations improve patient outcomes. Addressing these gaps is a research priority if XAI is to fulfill its potential as a facilitator of perioperative AI adoption.

## 1 Introduction

### 1.1 The rise of artificial intelligence in anesthesiology

Few environments in clinical medicine generate data at the density and temporal resolution of the modern operating theater. A single anesthetic case produces continuous, high-dimensional streams, arterial pressure waveforms, pharmacokinetic infusion records, EEG signals, ventilator parameters, intraoperative imaging, all simultaneously, at subsecond resolution. The anesthesiologist integrates some of this in real time; much of it scrolls past unprocessed. This is the informational landscape that has made perioperative medicine such fertile ground for artificial intelligence.

Over the past decade, the scope of AI applications in anesthesiology has expanded accordingly. Systematic reviews document ML and deep learning use across the full perioperative arc, preoperative risk stratification, intraoperative hemodynamic monitoring and drug titration, postoperative complication prediction (1). A 2024 meta-analysis in the British Journal of Anesthesia found that algorithm-driven management was associated with meaningful improvements in clinical outcomes across several RCTs (2), a transition from modelling exercises to interventional evidence that the field had been waiting for. Regulatory milestones followed: the Hypotension Prediction Index (HPI) received FDA clearance; AI-assisted ultrasound guidance for regional anesthesia obtained approval in several jurisdictions (3). The field had moved, in a decade, from proof-of-concept to regulatory approval. The technical case for ML-based prediction in anesthesiology has, in the span of a decade, moved from proof-of-concept to regulatory approval.

Prediction models are at the center of this effort. The clinical target list now spans post-induction hypotension (4–6), postoperative pulmonary complications (7, 8), AKI (9), delirium (10), MACCE (1), difficult airway (11–13), anesthesia depth (14–16), and postoperative pain (3, 17). Across these domains, ML-based models have demonstrated discrimination substantially exceeding conventional clinical scoring tools in multiple systematic reviews and meta-analyses (2, 18). The technical case for ML-based prediction in anesthesiology is now well established, supported by multiple systematic reviews, and increasingly, prospective interventional evidence.

What is in dispute, or should be, is what comes next.

### 1.2 Barriers to clinical translation: beyond the black box

Models can perform well and still sit untouched on the shelf. The gap between algorithmic performance and bedside adoption is real and well documented, but attributing it to a single cause oversimplifies a multifactorial problem. Three categories of barrier recur in implementation literature: failure to fit clinical workflows, absence of demonstrated outcome benefit, and limited interpretability (19–21).

Workflow integration is arguably the most immediate obstacle. An AI alert that arrives at the wrong moment in the clinical sequence, requires additional steps to act on, or generates frequent low-specificity warnings will be ignored regardless of its statistical performance. Cost and time considerations compound this: tools that add cognitive load without reducing workload or demonstrably improving outcomes face institutional resistance that accuracy metrics alone cannot overcome.

Interpretability is a distinct and increasingly prominent barrier, particularly for complex models. Most high-performing ML models, gradient boosted trees, random forests, deep neural networks, transform inputs into probabilities through computational mechanisms entirely opaque to the clinician who must act on them (19, 20). Unlike logistic regression or a decision tree, whose internal logic can be traced and audited, complex models offer no intelligible account of why a particular prediction was generated. A risk score without a rationale, without any indication of which features, in this patient, at this moment, are driving the alert, provides limited basis for targeted intervention, though it is worth noting that clinicians do act on opaque outputs in other contexts (pulse oximetry, BIS monitoring) when empirical evidence of benefit is sufficient.

In anesthesia specifically, opacity carries compounded risk under time pressure. Decisions must sometimes be made in minutes, an unrecognized difficult airway, an untreated hypotensive episode, an over-sedated patient deteriorating in the PACU. A clinician presented with a risk probability and no accompanying rationale can either trust the algorithm blindly or ignore it; neither option reflects what clinical decision support is meant to achieve (20).

Regulatory forces are now reinforcing what clinical common sense already demands. The EU AI Act (2024) classifies AI in medical decision support as high-risk, mandating transparency and explainability as foundational compliance requirements. The FDA’s Software as a Medical Device framework similarly requires interpretable, auditable performance characteristics. Black-box anesthesia models, however statistically impressive, face a narrowing path toward approval and widespread adoption.

As ML architectures grow more complex, more layers, more non-linear feature interactions, predictive accuracy tends to improve, but decision logic becomes correspondingly less accessible (20). That trade-off between performance and transparency is what has given rise to explainable artificial intelligence.

### 1.3 Explainable artificial intelligence: definition, dimensions, and strategic importance

Talking about XAI as if it were a single technique is one of the field’s more persistent confusions. In practice, it refers to a heterogeneous collection of tools, design principles, and evaluation approaches with a shared aim: making AI inputs, mechanisms, and outputs understandable to the humans who use them (21, 22). In clinical practice, “understandable” turns out to operate at several levels that are easily conflated but matter in quite different ways. A model can be transparent, its architecture and training process open to scrutiny, without being useful at the bedside. It can be interpretable, offering a traceable account of why a specific prediction was generated, and still require specialist AI knowledge to act on. Comprehensibility, the most demanding criterion, asks whether the explanation actually works in the hands of the intended clinical user under time-constrained conditions.

The TRIPOD+AI statement has elevated model interpretability to a core reporting requirement (23). Regulatory agencies and professional bodies have repositioned explainability from afterthought to design requirement. In anesthesiology, XAI-augmented models, most prominently using SHAP, LIME, attention mechanisms, and gradient-based visualization, have begun to generate feature attributions that align with established physiological knowledge and identify modifiable intraoperative targets (7, 21). Whether these mechanistic insights translate into measurably improved patient outcomes when embedded in clinical workflows is a different, and as yet, unanswered question: no prospective trial has directly compared AI-with-XAI against AI-alone on perioperative endpoints.

It is important to be clear about what XAI can and cannot claim. Interpretability is not a proven precondition for clinical adoption, the history of medicine includes many widely used tools whose mechanisms were poorly understood at the time of adoption (general anesthetic agents themselves being the most prominent example). Rather, XAI is increasingly expected by regulators, journals, and clinical governance bodies, and is postulated to facilitate adoption by enabling clinician oversight, supporting targeted intervention, and providing accountability. Whether it demonstrably improves patient outcomes compared with unaugmented prediction remains an open empirical question.

This review therefore has two aims: first, to critically synthesize the current evidence on XAI applications across five anesthesiology prediction domains, assessing not just whether prediction works but whether explanation adds something that prediction alone cannot deliver; and second, to identify the methodological and clinical research gaps that must be addressed before XAI-enhanced perioperative AI can be considered ready for routine practice.

### 1.4 Literature search strategy

To identify literature for this narrative synthesis, PubMed and Embase were searched from January 2018 to March 2026. These two databases were selected for their complementary coverage: PubMed provides comprehensive indexing of biomedical literature including MEDLINE, while Embase offers broader European and pharmacological coverage with less overlap. Search terms were combined using Boolean operators (AND/OR). XAI method descriptors included: “explainable artificial intelligence” OR “SHAP” OR “SHapley Additive exPlanations” OR “LIME” OR “attention mechanism” OR “GradCAM” OR “gradient-weighted class activation” OR “feature importance” OR “interpretable machine learning” OR “explainab*”. These were combined (AND) with anesthesiology domain terms: “anaesth*” OR “anesthes\*” OR “perioperative” OR “intraoperative” OR “postoperative” OR “airway” OR “analgesia” OR “sedation” OR “pain management.” Reference lists of included articles and relevant systematic reviews were hand-searched for additional studies.

Eligible studies were peer-reviewed original research, systematic reviews, or methodological papers that reported XAI analyses applied to anesthesiology prediction tasks, published in English, and involving human participants or clinical data. Conference abstracts, editorials, and studies applying AI without any XAI component were excluded. Following deduplication and iterative screening of titles, abstracts, and full texts, a subset of the retrieved literature was selected for inclusion in the narrative synthesis. Given the heterogeneity of XAI methods, outcome definitions, and clinical domains, the search is best characterised as targeted and informing rather than exhaustively systematic; a narrative synthesis was therefore performed, and no PROSPERO registration was sought.

## 2 XAI methodological framework

### 2.1 Taxonomy of XAI methods

The most clinically consequential distinction is between global and local explanation. Global methods characterize overall model behavior across a population, which features most consistently drive hypotension predictions across all patients. Local methods explain a single prediction, why this specific patient, at this moment, received a high-risk score. In anesthesiology, where decisions are irreducibly individual, local explanations carry the most practical weight. Global analyses remain valuable for a different purpose: if a model globally identifies features inconsistent with established physiology, that is a quality signal about the model itself.

Beyond scope, methods differ in their dependence on model architecture. Model-agnostic approaches, SHAP and LIME being the principal examples, can be applied post-hoc to any trained model, which matters when the best-performing model is a complex ensemble. Model-specific methods exploit internal structure, gradient flow in neural networks, attention weights in transformer architectures, and are therefore limited to particular model families, though typically more computationally efficient within them.

Explanation format is a separate dimension that tends to be underappreciated. Feature importance scores, counterfactual statements, rule extraction, and visual saliency maps serve different users and different clinical contexts. A SHAP waterfall plot may be informative to a researcher; a concise natural-language summary may be more compatible with active induction management. Format determines whether an explanation is usable in practice.

Table 1 provides a comparative matrix of the principal methods used in anesthesiology prediction research.

**Table 1:** Comparative overview of XAI methods applied in anesthesiology prediction research.

Method	Type	Scope	Model dependency	Computational cost	Primary anaesthesia applications	Key limitation
SHAP (SHapley Additive exPlanations)	Post-hoc model-agnostic	Global+Local	Model-agnostic (TreeSHAP for ensembles)	Medium (exact: exponential; TreeSHAP: linear)	Perioperative hypotension, PPC, POD, AKI, PONV, ICU sedation	Sensitive to feature correlation; may misattribute collinear predictors
LIME (Local Interpretable Model-agnostic Explanations)	Post-hoc model-agnostic	Local only	Model-agnostic	Low	Single-patient decision explanation; airway risk communication	Unstable: different sampling strategies produce divergent explanations
Attention Mechanisms	Intrinsic (architecture-specific)	Local (temporal)	Model-specific (Transformer/LSTM)	Low (computed during forward pass)	EEG-based depth monitoring; TCI drug infusion history; time-series prediction	Attention weight! = causal importance; may not reflect true feature relevance
GradCAM (Gradient-weighted Class Activation Mapping)	Post-hoc architecture-specific	Local (spatial)	Model-specific (CNN/DNN)	Low (single backward pass)	Difficult airway (facial imaging); ultrasound regional anaesthesia; ECG/EEG spectrograms	Limited to convolutional architectures; resolution degrades in deep networks
Intrinsically Interpretable Models (LR, DT, Nomogram)	Intrinsic transparency	Global (model logic is explanation)	N/A (model is its own explanation)	Minimal	Clinical scoring systems; preoperative risk stratification; regulatory-aligned decision support	May sacrifice 5–10% discrimination vs. complex models; assumes linearity/additivity

LR, logistic regression; CNN, convolutional neural network; EEG, electroencephalogram; TCI, target-controlled infusion; PPC, postoperative pulmonary complications; PONV, postoperative nausea and vomiting; AUROC, area under the receiver operating characteristic curve.

### 2.2 SHAP (SHapley additive exPlanations)

SHAP is the dominant XAI method in clinical anesthesia prediction research, appearing across hypotension, complication, sedation, and PONV prediction alike (7, 18, 24–27). Its mathematical foundations come from cooperative game theory: each feature is treated as a player in a coalitional game, and the SHAP value assigned to that feature represents its average marginal contribution to the prediction (39) across all possible feature coalitions. This Shapley-value-based decomposition satisfies formal properties, efficiency, symmetry, dummy, and additivity, that make it mathematically principled rather than heuristic (28).

SHAP generates explanations at two scales. Beeswarm and bar plots characterize which variables consistently matter across the whole cohort; waterfall and force plots decompose a specific patient’s risk score into the individual contributions of each feature, the output a clinician actually needs when making a decision about the patient in front of them.

Technical limitations deserve explicit attention. Exact Shapley computation scales exponentially with feature count; TreeSHAP and KernelSHAP provide efficient approximations with different trade-offs. More consequentially for physiological data: when predictors share substantial variance, serum creatinine and eGFR, systolic and mean arterial blood pressure, SHAP values can distribute attribution arbitrarily among collinear features, potentially misrepresenting which variable is independently clinically relevant.

### 2.3 LIME (local interpretable model-agnostic explanations)

LIME generates local explanations by fitting a simple linear surrogate to model responses near a given prediction (29). Its instability, small changes in perturbation sampling can produce materially different explanations from identical inputs, limits its suitability for high-stakes clinical decision support where reproducibility is a regulatory and ethical requirement. This instability may partly explain why SHAP has achieved broader adoption in the anesthesia literature. Nevertheless, LIME retains practical value as a rapid, model-agnostic local explanation tool in exploratory research settings, and its computational efficiency can be advantageous when near-real-time explanation is operationally required (Table 2).

**Table 2:** Summary of XAI evidence, clinical integration status, translational barriers, and maturity ratings across perioperative anesthesiology prediction domains. Evidence quality is based on study design and sample size at the time of review.

Clinical domain	Primary XAI methods	Evidence quality	Clinical integration	Key translational barriers	Representative references	Maturity rating
Intraoperative hypotension (PIH/IOH)	SHAP; Feature importance	Moderate-High (SR + meta)	Low (HPI deployed but opaque)	Feature instability across centres; HPI black-box not disclosed	Mohammadi 2024 Chen 2025 Katsin 2025	★★★☆☆
Postoperative pulmonary complications (PPC)	SHAP; XGBoost explanation	Moderate-High (prospective)	Low (web tool under validation)	Lack of multi-centre SHAP stability data	Li 2024 Xu 2025	★★★☆☆
Postoperative delirium (POD)	Feature importance; SHAP (limited)	Moderate (SR; few XAI studies)	Very Low	Majority of models report no XAI analysis	Tu 2025 Wan 2025	★☆☆☆☆
Acute kidney injury (AKI)	Feature importance (SHAP limited)	Moderate (large multi-centre cohort)	Low (web predictor deployed)	No patient-level XAI explanation implemented	Min 2025	★★☆☆☆
Major adverse CV events (MACCE)	Attention mechanism; multimodal	Moderate (single centre)	Very Low	Multimodal explanation integration complex	(see text)	★★☆☆☆
Difficult airway prediction	GradCAM; Multiparametric SHAP	Low-Moderate (SR; no prosp. XAI outcome)	Low (commercial AI widely deployed)	Demographic bias; GradCAM equity audit absent	Abosamak 2025 Sorbello 2026	★★☆☆☆
Depth of anaesthesia/BIS monitoring	Attention; SHAP on EEG features	Moderate (multi-agent; bothways valid.)	Low (BIS widely used; black box)	Open models lack prospective clinical evaluation	Hwang 2023 Wang 2025	★★★☆☆
Regional anaesthesia & ultrasound AI	GradCAM overlays	Low (FDA approved; limited XAI)	Moderate (FDA-cleared product)	Commercial XAI not publicly documented	Adams 2025 Schroeder 2025	★★☆☆☆
PONV prediction	SHAP; Feature importance	Moderate (multiple ML studies)	Low	Lack of prospective validation	Chang 2026	★★★☆☆
ICU sedation & pain management	SHAP; Ensemble explanation	Low-Moderate	Very Low	Data fragmentation; weak XAI reporting	Dai 2025	★☆☆☆☆

Maturity Rating key, ★★★★★: multi-centre prospective validation + outcome evidence; ★★★★☆: prospective cohort + local SHAP + clinical correlation; ★★★☆☆: retrospective cohort + global & local SHAP + physiological interpretation; ★★☆☆☆: feature importance reported, limited clinical interpretation; ★☆☆☆☆: minimal or no XAI analysis. SR, systematic review; CV, cardiovascular; PONV, postoperative nausea and vomiting; ICU, intensive care unit; BIS, bispectral index.

### 2.4 Attention mechanisms as an intrinsic XAI tool

Attention weights are not feature importance scores, a conflation common in clinical AI literature whose consequences are not trivial. Jain and Wallace (30) demonstrated that attention distributions can be manipulated without changing model predictions, and that entirely different attention configurations can achieve identical predictive performance. An anesthesiologist who interprets a high attention weight at a particular EEG time step as evidence that moment caused the predicted anesthesia depth is drawing an inference the mathematics does not support.

That said, attention mechanisms do provide genuine interpretability for temporal physiological data. Hwang et al. BIS prediction model (17) showed that higher-frequency EEG amplitude components were most associated with lighter anesthesia, while lower-frequency *δ*-range oscillations received greater weight at lower predicted BIS, consistent with established neurophysiology of anesthetic-induced cortical suppression. Wang et al. hybrid LSTM–Transformer (20) extended the principle to pharmacology, identifying which preceding infusion time steps most influenced current depth estimates.

### 2.5 Gradient-based visualization methods (GradCAM and variants)

For models processing structured spatial data, medical images, ultrasound frames, EEG spectrograms, GradCAM computes the gradient of the target class score with respect to convolutional feature maps, producing a spatial heatmap that highlights which regions most strongly activated the prediction. In anesthesiology, its value is most direct for imaging-based airway assessment and ultrasound guidance: when a model’s attention concentrates on anatomically meaningful structures, the heatmap provides construct validation; when it attends to image artefacts, it reveals training data confounds invisible to AUC-based reporting (12, 13). It is important to note that anatomical plausibility of a GradCAM heatmap does not guarantee faithfulness to the model’s internal decision process: a heatmap may highlight physiologically sensible regions while still imperfectly representing the computational pathway that generated the prediction.

### 2.6 Intrinsically interpretable models

Before reaching for SHAP or GradCAM, it is worth asking whether a given problem actually requires a black box at all. Logistic regression, decision trees, and nomograms are interpretable by construction, and several recent anesthesia studies have found LASSO-penalised logistic regression approaching or matching complex ensemble performance (6, 8, 9, 19). Hybrid strategies, deploying a complex model for prediction while presenting a simplified logistic regression approximation to the clinician, represent a pragmatic middle ground with growing uptake (22).

## 3 XAI in perioperative hypotension prediction

### 3.1 Clinical background and the predictive challenge

Intraoperative hypotension, conventionally defined as MAP below 65 mmHg, has reported incidences ranging from 5% to over 40% depending on surgical population and threshold applied. Post-induction hypotension is of particular concern: vasodilatory and myocardial-depressant effects of induction agents compound pre-existing cardiovascular vulnerability precisely when compensatory capacity is most constrained, with sustained MAP depression independently associated with myocardial injury, AKI, cerebrovascular events, and increased 30-day mortality (6). Despite multiple ML models substantially outperforming conventional risk scores, a clinically actionable explanation of why a specific patient is high-risk has remained elusive without XAI.

### 3.2 Machine learning models and XAI-generated clinical value

Chen and Zhang (5) applied four model types to 5,406 patients; SHAP analysis identified baseline systolic and diastolic blood pressure, age, sex, surgery type, and specific induction agents as primary risk drivers. Katsin et al. (7) worked with 20,309 patients across non-obstetric surgeries, PIH occurred in 24.4% of cases. Their SHAP analysis picked out pre-induction systolic blood pressure, pre-induction MAP, propofol dose, and beta-blocker use as dominant predictors (AUC 0.732).

Consider what “PIH probability: 78%” actually tells a clinician in isolation. Nothing about which factors are driving it. Nothing about what to change. The SHAP analyses across these studies (5, 7) consistently translate probabilities into specific, modifiable targets: when pre-induction MAP, propofol equivalent dose, and beta-blocker or ACEI use are identified as dominant risk contributors, the clinical implications become hypothesis-generating rather than abstract, directing attention towards preoperative haemodynamic optimisation, induction agent dosing, and prophylactic vasopressor consideration. These are directions for clinical reasoning, not treatment directives derived directly from SHAP values; whether acting on such explanations improves outcomes requires prospective validation. A bare probability number does not generate even these starting points; the feature-level explanation does.

The commercially deployed HPI device illustrates the contrasting case. The system has demonstrated efficacy in RCTs for reducing total hypotension duration (6), and it is in daily use in many centers. But its algorithm is proprietary and undisclosed. Clinicians cannot inspect which features drive a specific alert; they cannot audit the system’s behavior in atypical cases. The absence of explainability likely contributes to wide variability in HPI adoption across departments, and in an era of increasingly stringent AI governance, that accountability gap will become harder to defend.

### 3.3 Current limitations

SHAP values describe correlational contributions within a trained predictive model, not causal pathways. The fact that pre-induction MAP is the strongest SHAP predictor does not mean that artificially elevating MAP before induction would proportionally reduce PIH risk. The distinction between “this variable predicts the outcome” and “changing this variable will change the outcome” is fundamental, and is currently underemphasized in much of the literature.

## 4 XAI in perioperative complication prediction

### 4.1 Postoperative pulmonary complications: the most developed XAI application

Li et al. 2024 study in the British Journal of Anesthesia (9) demonstrates what the field is capable of when XAI is treated as a primary scientific contribution rather than a reporting addendum.

The study analysed 10,284 patients aged 65 and older, incorporating intraoperative respiratory dynamic variables, tidal volume, peak inspiratory pressure, PEEP, plateau pressure, compliance, and driving pressure, processed as time-series summaries. An optimized XGBoost achieved AUROCs of 0.878 and 0.881 in validation and prospective cohorts, against an ARISCAT AUROC of 0.496–0.533. The SHAP analysis identified respiratory system dynamic compliance, mechanical power, and driving pressure as the three leading modifiable intraoperative risk contributors, variables under direct anesthesiologist control, not fixed patient characteristics. That the model arrived at these targets from observational data alone, without being told anything about the physiology of ventilator-induced lung injury, is consistent with the hypothesis that the model has captured clinically meaningful physiological signal rather than statistical noise, though causal attribution requires prospective validation (31). A 20-variable simplified model (AUROC 0.864) is now deployed as a web-based clinical tool.

A multicenter validation study (10) involving four hospitals and neurosurgical patients raised an issue worth noting: features statistically significant in multivariate logistic regression did not fully overlap with those assigned highest SHAP values. Traditional statistical importance and XAI-derived importance measure different things; which is more clinically meaningful depends on what question you are trying to answer.

### 4.2 Postoperative delirium: an underserved domain for XAI

Postoperative delirium is simultaneously one of the most clinically consequential and least XAI-developed prediction targets in perioperative medicine. POD affects 10–60% of surgical patients, with incidence rising sharply in elderly patients undergoing cardiac, major vascular, or prolonged abdominal procedures, and is independently associated with prolonged ICU stays, accelerated cognitive decline, increased one-year mortality, and substantial carer burden (11). A 2025 meta-analysis synthesized 17 studies and 69 ML prediction models across 2,05,202 individuals, reporting a combined AUROC of 0.83 (11). The discriminative performance is credible. The critical limitation is that the majority of these models reported no XAI analysis. This matters in a way that it does not for hypotension prediction. The pathophysiology of POD involves overlapping neuroinflammatory, cholinergic, and circadian mechanisms; the modifiable perioperative risk factors are numerous, clinician-controllable, and intervention-responsive: anticholinergic drug burden, sleep disruption, pain control strategy, intraoperative depth of anaesthesia, and haemodynamic management are all candidate targets. An XAI-enhanced POD prediction model could, in principle, distinguish between risk driven primarily by fixed factors (age, pre-existing cognitive impairment, comorbidity burden) and risk driven by modifiable intraoperative factors, a clinically decisive distinction that a bare probability score cannot provide. That this XAI infrastructure is almost entirely absent from the published literature represents the largest unrealised opportunity in perioperative AI.

The exception that shows what is possible: Wan et al. (12) applied SHAP combined with mediation analysis in 555 patients undergoing radical colorectal surgery. The dominant contributors turned out to be lipid profile variables, total cholesterol, triglycerides, and trimethylamine-N-oxide, with mediation analysis establishing that these biomarkers partially mediate the pathway from gut microbiome dysregulation to POD. This is mechanistic hypothesis generation, not just feature attribution. The gap between that standard and where most of the literature sits is large.

### 4.3 Postoperative AKI and MACCE

The CMC-AKIX model (1), 2,39,267 surgeries across seven Korean university hospitals, is the largest scale ML-based AKI prediction effort reviewed here. Feature importance pointed to preoperative renal function, intraoperative hemodynamic instability, and surgical duration as leading risk determinants, consistent with established pathophysiology. However, without patient-level local SHAP explanations, the tool cannot communicate the clinically essential distinction: is elevated risk driven primarily by pre-existing CKD (grounds for heightened surveillance) or anticipated hemodynamic instability (grounds for aggressive goal-directed therapy)? Future AKI prediction tools should treat patient-level explanation as a design requirement from the outset.

MACCE prediction represents the most important XAI gap in perioperative cardiac risk assessment. Several ML models have demonstrated good discrimination for MACCE, integrating demographics, ICD-10 diagnoses, and ECG-derived features, but published validation studies in this domain have not paired their predictive algorithms with systematic local explainability analyses. Until patient-level SHAP or attention visualization is applied to well-validated MACCE models, it remains impossible to determine whether the ECG morphological features that drive model predictions are clinically interpretable or merely statistical artefacts of training data composition.

## 5 XAI in difficult airway management

### 5.1 Clinical urgency and the limits of conventional assessment

The “cannot intubate, cannot oxygenate” scenario represents the highest-stakes decision in anesthesia. Conventional bedside prediction tools, Mallampati classification, thyromental distance, mouth opening, neck range of motion, are individually characterized by sensitivities in the 30–60% range and low positive predictive values. Experienced airway assessors integrate these and additional cues through clinical pattern recognition, but that expertise is neither uniformly distributed nor reliably available for patients with obesity, previous radiotherapy to the neck, or craniofacial anomalies where standard prediction rules do not apply.

A systematic review and meta-analysis (14) synthesized 13 studies, finding deep learning models achieving AUROCs up to 0.84 and traditional ML around 0.80–0.81. A 2026 narrative review in the British Journal of Anesthesia (13) explicitly identifies the black-box nature of some AI algorithms as a key barrier to clinical acceptance, coming from a clinical review rather than a methodology paper, that judgement is more consequential: explainability is a practical facilitator for airway AI reaching the operating theater.

### 5.2 XAI applications in imaging-based airway prediction

CNNs trained on facial photographs or lateral neck images represent the dominant AI modality for difficult airway prediction, detecting anatomical predictors that the human eye may not reliably quantify (12). For these models, GradCAM is the natural explanatory tool.

The question GradCAM answers is clinically direct: where is the model looking when it flags this patient? When the generated saliency map concentrates on mandibular contour, thyroid prominence, neck circumference, and hyoid position, the anatomy an experienced airway assessor would attend to, the model is behaving credibly, and the heatmap serves as construct validation (12, 13). When it highlights background texture or image compression artefacts, the model has learned to use features with no physiological basis for predicting laryngoscopy difficulty. AUC-based reporting would never surface this; GradCAM does, before the model goes near a patient.

The bias-detection function is particularly consequential given documented concerns about differential AI performance across demographic groups when training datasets are predominantly derived from homogeneous patient populations. An equity audit question, does model attention differ systematically by patient demographic characteristics?, is one that GradCAM can directly address. It has not been routinely performed in published literature, and should be.

### 5.3 Clinical translation: from screening to procedural guidance

AI applications in airway management are extending beyond preoperative prediction into the procedure itself. AI-powered video-laryngoscopy systems now offer real-time anatomical structure identification and tracheal tube placement verification through image-based depth estimation (13).

**Figure 1:**
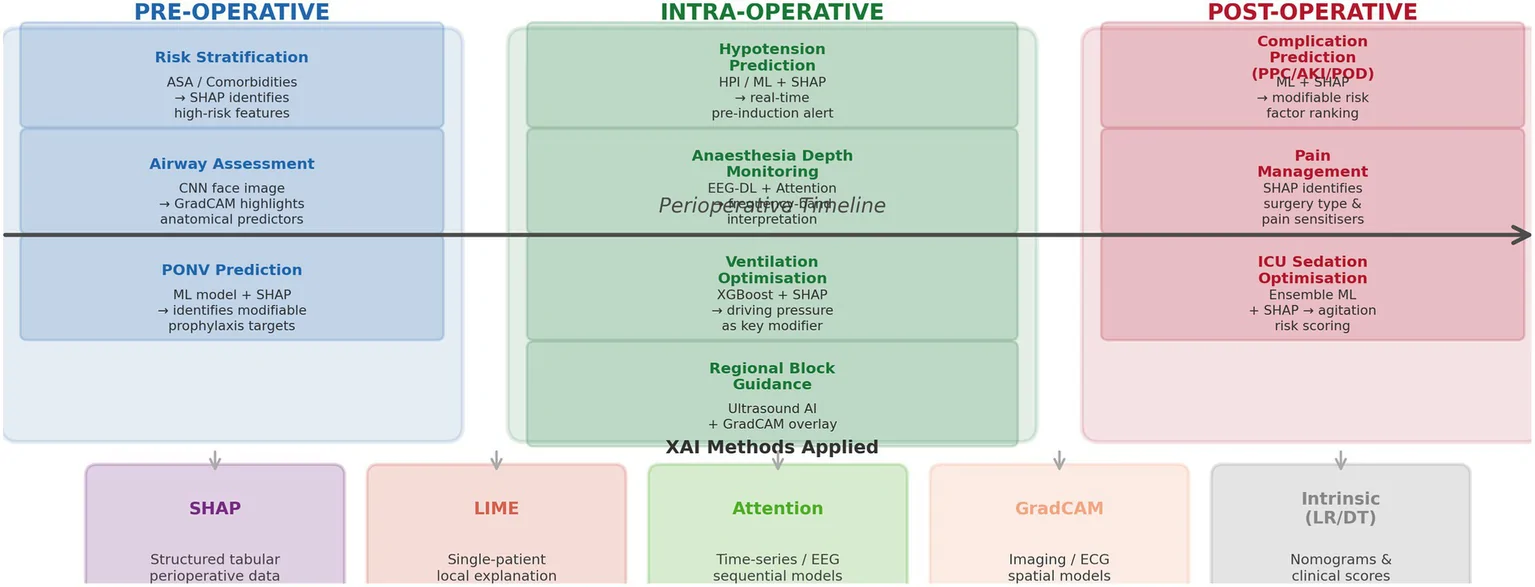
Explainable AI across the perioperative continuum. Three phases of care (pre-operative, intra-operative, post-operative) are shown with their respective XAI-augmented prediction applications. The five principal XAI methods and their primary data domains are illustrated in the lower panel.

In this procedural context, the XAI question shifts from pre-operative risk stratification to something more immediate: which visual features is the model using to identify the vocal cords right now? Real-time GradCAM overlays could flag discordance between AI attention and the operator’s own anatomical recognition before tube advancement, a genuinely different use case that existing research has yet to systematically address (Figure 1).

## 6 XAI in depth of anesthesia monitoring and target-controlled infusion

### 6.1 From proprietary black boxes to interpretable open models

**Figure 2:**
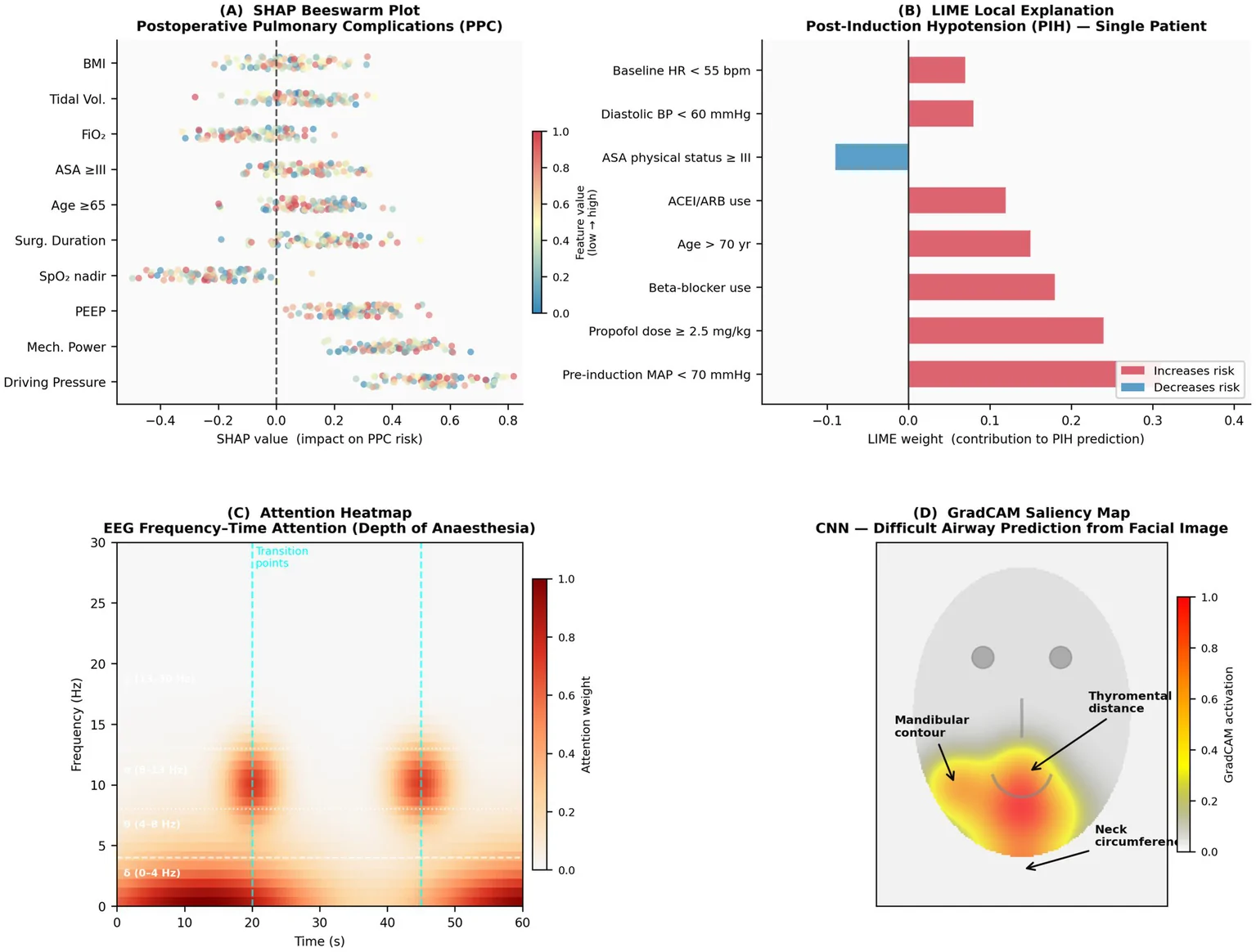
[All panels: Simulated/schematic data for illustration only] Representative XAI visualizations across anesthesiology prediction domains. **(A)** SHAP beeswarm plot for postoperative pulmonary complication (PPC) prediction: Each point represents one patient; color indicates feature value (blue = low, red = high). **(B)** LIME local explanation for post-induction hypotension (PIH) in a single patient: Bar length reflects feature contribution to the predicted risk. **(C)** Attention heatmap from a Transformer-based depth-of-anaesthesia model: Warm colors indicate high attention to specific EEG frequency-time regions at state-transition boundaries. **(D)** GradCAM saliency map superimposed on a facial image for difficult-airway classification: Activation concentrates on mandibular contour, thyromental region, and neck circumference. All panels are schematic/simulated illustrations based on published model architectures.

The history of AI in anesthesiology has an irony that rarely surfaces in the literature. The Bispectral Index, the field’s first commercially successful AI device, generates its 0–100 score through a proprietary, undisclosed computational pipeline. Clinicians adopted it through accumulated empirical experience rather than mechanistic understanding, developing workarounds for known failure modes, burst suppression in elderly patients, ketamine-induced BIS elevation, EMG artefact confounding, without ever being able to inspect the underlying logic. Clinical adoption of opaque AI is possible when a tool has been validated through extensive prospective trials demonstrating patient outcome benefit, BIS earned clinical trust through accumulated RCT evidence even without algorithmic transparency. The distinction between clinical validation and algorithmic transparency matters: clinical validation establishes that a tool improves outcomes; algorithmic transparency explains why, and enables rational troubleshooting when the tool behaves unexpectedly. BIS illustrates that the two can be decoupled, but also that decoupling has costs. Without access to the underlying computation, each failure mode (ketamine-induced BIS elevation, EMG artefact confounding, burst suppression in elderly patients) had to be discovered empirically rather than anticipated from the model architecture. New AI tools for anaesthesia should aim for both: prospective outcome evidence and interpretable computation, rather than treating them as alternatives (Figure 2).

Hwang et al. (17) explicitly framed their work as a response to this limitation. They decomposed 30-s EEG windows into amplitude and phase components via Fast Fourier Transform, then applied an attention mechanism over these frequency-domain components. The model predicted continuous BIS values 25 s ahead (RMSE 6.614) and achieved AUROC 0.937 for binary classification. The interpretability analysis showed that higher-frequency EEG amplitude components were most associated with lighter anesthesia, while lower-frequency *δ*-range oscillations received greater attention weight at lower predicted BIS, consistent with decades of neurophysiological research on anesthetic-induced cortical suppression.

### 6.2 ML for depth monitoring across diverse agents and populations

Most EEG-based depth monitoring algorithms have been developed and validated on propofol data. Extending these to other agents is non-trivial: esketamine induces a dissociative state with gamma oscillations that look nothing like propofol-induced spindle activity. Tu et al. (16) applied ML-based EEG depth monitoring to both propofol and esketamine, using a novel intra-subject dataset partitioning approach to assess cross-individual generalisation, the prerequisite for clinical deployment.

The paediatric case is harder still. A separate investigation (22) applied quantitative EEG features with ML to predict expired sevoflurane concentration in young infants, a population where BIS calibration based on adult data is unreliable because infant EEG maturation produces fundamentally different spectral patterns. Feature importance analysis distinguished the qEEG spectral parameters that differentiate infant from adult EEG during sevoflurane administration, providing a mechanistic basis for the conclusion that depth monitoring tools trained on adult data are not safely extrapolated downward.

### 6.3 Closed-loop systems and the XAI imperative

The hybrid LSTM–Transformer model by Wang et al. (20) achieves ML-enhanced target-controlled infusion, with Transformer attention output providing temporal XAI that identifies which preceding time steps in the infusion history most influenced current depth predictions, making visible the pharmacodynamic assumptions encoded in the model’s learned weights.

In a closed-loop system that automatically adjusts propofol infusion based on a continuous physiological feedback signal, the supervising anesthesiologist’s moment-to-moment involvement in drug titration is reduced. Systematic reviews have confirmed that ML-based automated drug titration and real-time physiological optimization are technically feasible across diverse clinical scenarios (37). But that reduction correspondingly diminishes the ability to catch algorithmic errors. If the closed-loop controller is responding to an EEG sensor artefact rather than genuine depth change, and the XAI output cannot distinguish the two, the supervising clinician has no basis for intervention. In closed-loop anesthesia delivery, explainability is the mechanism through which nominal supervision becomes real supervision.

## 7 XAI in regional anesthesia and pain management

### 7.1 AI in ultrasound-guided regional anesthesia

Ultrasound-guided regional anesthesia has seen rapid growth in AI-assisted applications over the past five years. Deep learning models for real-time anatomical structure recognition, identifying the brachial plexus, sciatic nerve, and perineural sheath on live ultrasound, have been developed and, in at least one commercial system, received regulatory approval (3). For these models, GradCAM-type visualization can highlight identified structures on the live display, providing a second-opinion function that may be particularly valuable for trainees or non-specialists performing blocks under time pressure in resource-limited settings (17).

Several studies have examined AI-assisted nerve identification in ultrasound-guided blocks, reporting improved identification accuracy and reduced time to block performance (32). Automated needle tip tracking and nerve segmentation systems using deep learning have demonstrated feasibility in clinical settings, with attention-based visualization confirming that model focus concentrates on anatomically relevant structures (33, 34). The explainability properties of commercially deployed systems, however, remain largely undocumented in the public literature, a gap that warrants attention as these tools move toward routine clinical use (35, 38).

### 7.2 Postoperative nausea and vomiting and pain prediction

In the PONV prediction study by Chang et al. (36), SHAP analysis assigned strongly negative values to dexamethasone use, identifying it as a protective factor, consistently reducing predicted PONV risk. This aligns exactly with dexamethasone’s established anti-emetic mechanism and guideline-recommended prophylactic use in high-risk patients. When XAI output tracks this cleanly with guideline-level clinical evidence, it provides reassurance that the model has learned the relevant biology rather than statistical noise from an imbalanced training set.

In postoperative pain prediction, SHAP analysis has consistently pointed to surgery type, baseline pain scores, preoperative opioid tolerance, and psychosocial factors as leading predictors of inadequate analgesia and opioid-related adverse events (3, 18, 27). These attributions are clinically actionable: they identify patients most likely to benefit from perioperative multimodal analgesia optimisation, pain psychology consultation, or early specialist acute pain team involvement, the kind of resource allocation guidance that standard population-level protocols cannot provide.

### 7.3 Current state of XAI in this domain

**Figure 3:**
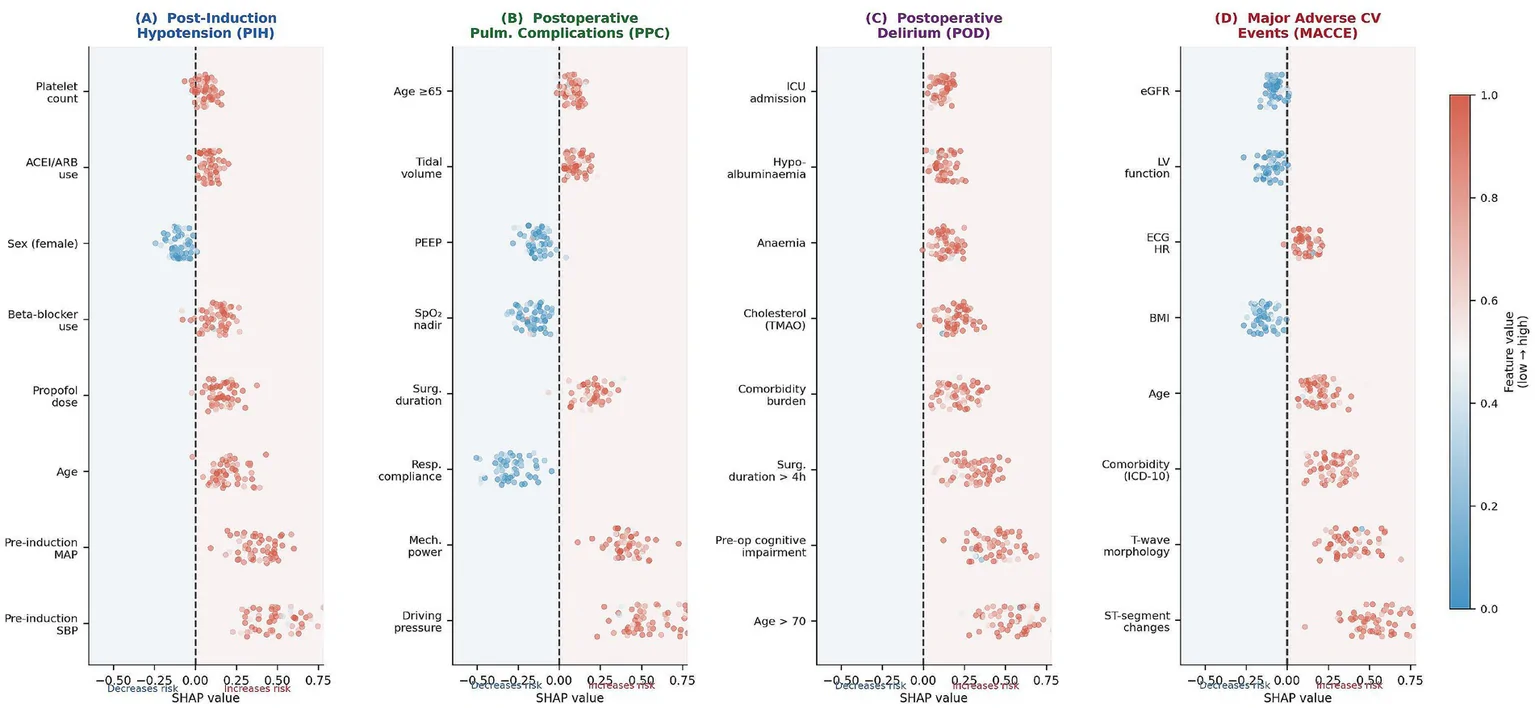
[All data simulated based on published feature importance rankings] SHAP beeswarm plots for four perioperative complication domains. **(A)** Post-induction hypotension (PIH): Pre-induction blood pressure parameters dominate. **(B)** Postoperative pulmonary complications (PPC): Modifiable ventilatory parameters (driving pressure, mechanical power, compliance) lead the attribution. **(C)** Postoperative delirium (POD): Non-modifiable demographic factors and metabolic biomarkers predominate. **(D)** Major adverse cardiac and cerebrovascular events (MACCE): ECG-derived features (ST-segment and T-wave morphology) emerge alongside clinical comorbidities. Colour gradient (blue to red) represents feature values (low to high). Distributions are simulated based on published feature importance rankings.

Regional anesthesia and pain management is the least XAI-developed of the five domains reviewed. Available studies are largely single-center, and XAI analyses consist primarily of global SHAP rankings rather than patient-level explanations. The procedural XAI question, which anatomical features is the model using to identify the target structure in real time?, is technically tractable but remains underexplored. Pain prediction faces an additional complexity in that outcome operationalisation (analgesic adequacy, chronic pain transition) varies widely across studies, limiting comparability of XAI-derived attributions (Figure 3).

## 8 Challenges, limitations, and barriers to clinical translation

### 8.1 Technical limitations of current XAI methods

**Figure 4:**
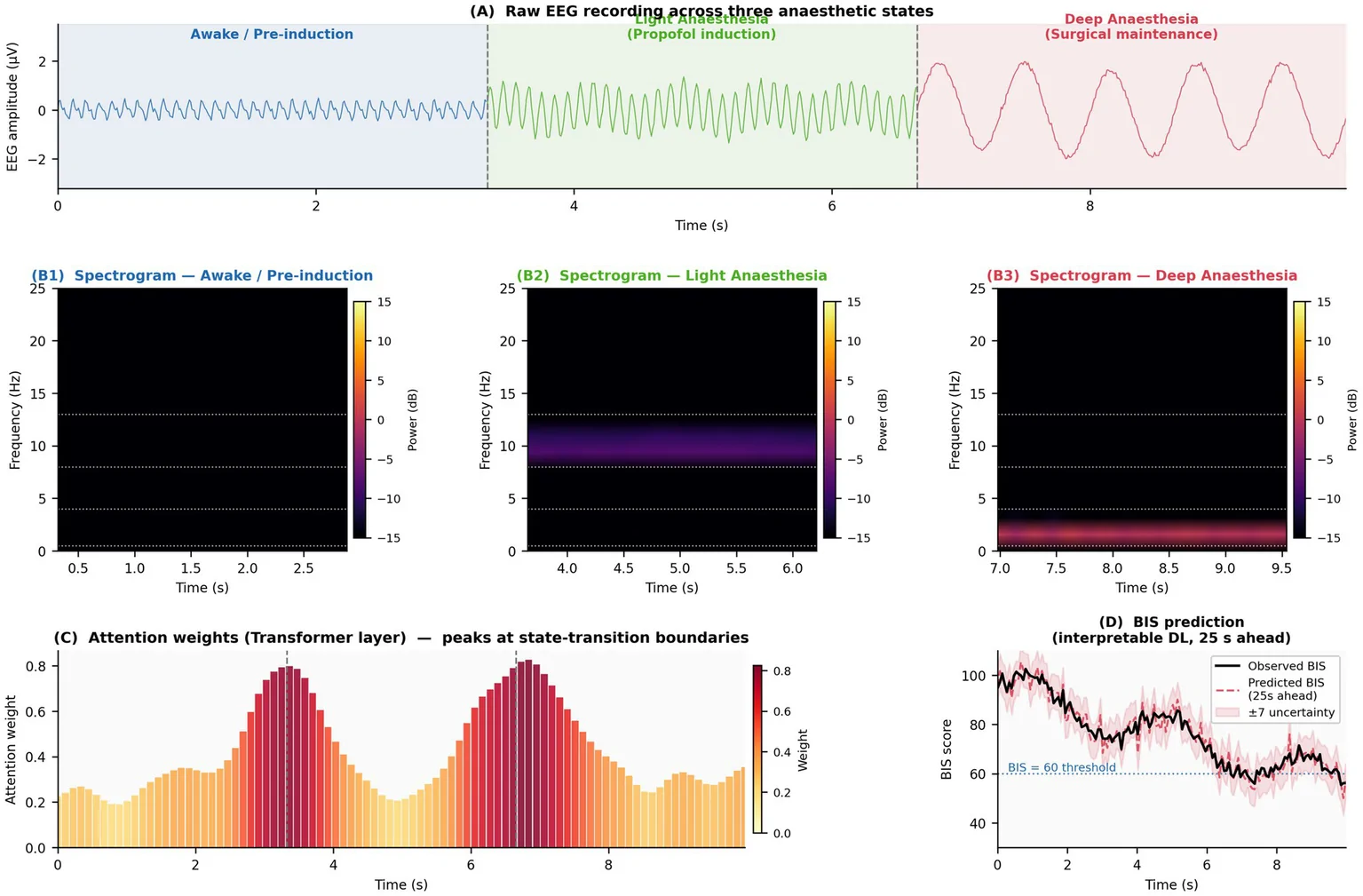
[All data simulated for illustrative purposes] EEG-based XAI for depth-of-anaesthesia monitoring. **(A)** Simulated raw EEG recording across three anaesthetic states: Awake (dominant alpha, 8–13 Hz), light anaesthesia (alpha-delta transition), and deep anaesthesia (dominant delta, 0–4 Hz). **(B1–B3)** Time-frequency spectrograms for each state, illustrating the progressive shift in spectral power from alpha/beta to delta frequencies with increasing anaesthetic depth. Horizontal dashed lines demarcate canonical EEG frequency bands. **(C)** Attention weight distribution from a Transformer encoder layer: Peaks coincide with state-transition boundaries, providing a temporal interpretability signal for the depth-prediction model. **(D)** BIS score prediction (25 s ahead) by an interpretable deep-learning model; predicted values (dashed red) closely track observed BIS (solid black) with uncertainty bounds indicated. All data are simulated for illustrative purposes.

Stenwig et al. (26) compared XAI methods across multiple ML models for ICU mortality prediction and found that SHAP-derived feature importance rankings frequently diverged both from established clinical knowledge and from each other, concluding that “calibration and impact of individual features differed considerably and did in multiple cases not correspond to common medical theory”. Models with high AUROCs were generating explanations that experienced clinicians would recognize as physiologically implausible (Figure 4).

The implications are uncomfortable. If explanation validity does not follow from predictive performance, clinicians cannot use discriminatory accuracy as a proxy for the trustworthiness of model explanations. Each requires independent evaluation, yet the methods and benchmarks for assessing explanation quality are far less developed than those for assessing prediction performance.

SHAP has specific vulnerabilities for physiological data: collinear predictors receive arbitrarily distributed attribution, potentially misrepresenting which variable is independently relevant. LIME is worse, its local linear approximation produces materially different attributions from identical inputs under different random seeds. Approximate Shapley methods (TreeSHAP, KernelSHAP) reduce computation time at the cost of approximation error, creating practical tension with real-time monitoring requirements.

### 8.2 The absence of standardized evaluation frameworks

There is a structural mismatch at the heart of the field. Prediction model performance evaluation has clear, well-established standards: AUROC for discrimination, Hosmer-Lemeshow for calibration, decision curve analysis for clinical utility. For XAI outputs, the explanations that are supposed to make AI clinically usable, no analogous consensus framework exists.

Such a framework would need to operationalize at least four properties with specific, measurable criteria. Fidelity to internal model logic can be quantified by training a surrogate model on SHAP-selected features and comparing its predictions against the full model on held-out data, a measurable agreement metric whose acceptable threshold requires empirical determination in the clinical context. Stability of explanations across similar inputs can be assessed by calculating the rank-order correlation of SHAP feature attributions under repeated small input perturbations, providing a reproducibility index that could be reported alongside model performance metrics. Comprehensibility to the intended clinical user requires prospective evaluation with validated decision-support instruments administered to anesthesiologists rather than data scientists, the System Usability Scale and domain-specific cognitive load measures are available candidates. Clinical alignment with established pathophysiological knowledge can be evaluated by structured expert elicitation: panels of experienced clinicians rating whether the top-ranked SHAP features are physiologically plausible and clinically actionable, with agreement criteria defined *a priori*. Establishing acceptable thresholds for each of these metrics requires dedicated methodological work and cross-institutional validation; none currently exist for XAI in anaesthesia.

The TRIPOD+AI statement addresses interpretability in its reporting guidelines (23) but remains relatively general on XAI-specific requirements. As a result, XAI reporting in anesthesia prediction literature ranges from a single global feature importance bar chart to comprehensive local SHAP analyses with physiological correlation, contributions that are not equivalent, but for which there is no accepted standard of comparison.

### 8.3 Implementation, regulatory, and liability considerations

The interface design problem is routinely underestimated. Building a SHAP analysis that runs in near-real time is technically solvable; building a presentation that a busy anesthesiologist will look at, correctly interpret, and act on during induction, in the thirty seconds between propofol and first incision, is a human-factors challenge that has received almost no formal research attention. Alert fatigue applies directly: low-specificity XAI alerts will, over time, be ignored. Underlying all of this is a data infrastructure problem: AIMS quality and standardization vary considerably across institutions, and EHR integration requires governance approval and workflow redesign that algorithms alone cannot address.

On the regulatory side, the EU AI Act (2024) classifies AI-assisted clinical decision support as high-risk and requires documentation of model logic and human oversight mechanisms, but does not mandate specific XAI methods or patient-level feature attributions such as individual SHAP decompositions. Similarly, the FDA’s Software as a Medical Device (SaMD) framework requires transparent and auditable performance characteristics without prescribing particular explanation formats. Neither framework yet specifies minimum XAI technical requirements for anaesthesia decision support. Simultaneously, legal accountability chains, from model output through XAI explanation to clinical decision to patient harm, remain undefined in most jurisdictions, actively discouraging institutional adoption. The gap between regulatory intent and regulatory implementation creates uncertainty that the field cannot afford to leave unresolved.

## 9 Future directions and research agenda

### 9.1 Methodological innovations

Among near-term priorities, large language model integration may be the most practically consequential, not for its methodological sophistication, but for the specific barrier it attacks: translating XAI outputs into a form clinicians can act on. A waterfall plot of SHAP values is informative to a data scientist but cognitively demanding during induction management. Converting the same information into a natural-language summary, specifying which features drive elevated risk and what adjustments follow, could make XAI usable without specialist AI literacy. However, LLM integration introduces its own risk profile that must be explicitly managed. Hallucination, the generation of fluent but factually incorrect statements, is a documented LLM failure mode that, in a perioperative decision-support context, could produce confidently stated but clinically dangerous recommendations. Constrained generation approaches, in which LLM output is anchored to verified SHAP values and predefined clinical knowledge bases rather than unconstrained generation, are a necessary architectural safeguard. Provenance tracking, recording which SHAP features, threshold values, and knowledge-based entries contributed to each LLM-produced statement, is equally essential for audit, regulatory compliance, and clinician trust. LLM-enhanced XAI tools that lack these safeguards may produce explanations that are readable but unreliable, potentially a worse outcome than presenting the raw SHAP output.

The deeper methodological gap is in causal XAI. Current SHAP-based explanations identify feature contributions within a trained predictive model, a correlational, model-internal operation. The clinically pressing question is different: if we modify this intraoperative variable, what change in outcome can we expect? Counterfactual explanation methods, generating the minimal feature changes needed to shift a prediction from high-risk to low-risk, offer a partial bridge, and represent the direction most likely to close the gap between feature attribution and clinically actionable recommendations.

### 9.2 Clinical research agenda

The most uncomfortable gap in the XAI anesthesia literature has nothing to do with methods. Whether XAI-enhanced AI recommendations lead to different and better clinical decisions than AI predictions alone, this question has simply not been asked in a prospective trial. Not once.

The gap is closable. Randomised controlled trials comparing AI prediction alone versus AI prediction plus XAI explanation versus standard clinical assessment on patient-level outcomes, hypotension incidence, PPCs, PONV, length of stay, are feasible within existing large anesthesia research networks. Intermediate endpoints are measurable: rate of propofol dose modification in high-PIH-risk patients, clinician confidence in AI recommendations, adherence to protocol changes suggested by the AI system. Until these trials are done, any claim that XAI adds clinical value rather than research value rests on inference, not evidence.

A related priority: multicenter external validation of XAI explanations, not just prediction model discrimination. If SHAP identifies pre-induction MAP and beta-blocker use as dominant PIH predictors in a South Korean academic center but not in a European private hospital, that instability has direct implications for the safety of globally deployed anesthesia AI tools.

### 9.3 Equity, ethics, and standardization

A model that performs well on average can fail specific subgroups, and an XAI tool generating plausible explanations for the majority may generate misleading ones for patients outside the training distribution. Demographic subgroup analysis of SHAP feature attributions should become standard practice. Where differential attributions are found, the interpretation requires care: age-related pharmacokinetic differences and sex differences in hemodynamic response to induction are real, and differential attribution may reflect genuine biology or training data imbalance, two explanations that call for entirely different corrective responses.

## 10 Conclusion

Machine learning prediction models in anesthesiology have made a real and substantial contribution. Across hypotension, pulmonary complications, delirium, difficult airway, and anesthesia depth, algorithmic approaches consistently outperform conventional clinical scoring tools, and the evidence base is now broad enough to be credible rather than merely promising.

The central argument of this review is that this contribution remains largely unrealised at the bedside, and that the missing elements include not only explanation, but also workflow integration, demonstrated outcome benefit, and institutional infrastructure. Anesthesiologists are not passive recipients of algorithmic outputs; they are accountable professionals who must integrate AI recommendations with clinical judgement, institutional context, and direct patient assessment. That integration is facilitated by explanations. Without them, the rational response to an AI alert, whatever its accuracy, is scepticism, and the result, over and over, is the adoption gap that has frustrated this field for a decade. Whether XAI alone is sufficient to close that gap, or whether it is necessary but not sufficient alongside prospective outcome evidence, workflow integration, and institutional infrastructure, remains an open empirical question.

The current XAI landscape is uneven. SHAP has achieved meaningful uptake in hypotension and complication prediction, generating actionable feature attributions that align with physiological knowledge. Attention mechanisms and GradCAM have produced interpretable insights into EEG-based depth monitoring and airway assessment. These are genuine achievements. But most delirium prediction models lack any XAI analysis; no standardized evaluation framework exists for XAI outputs; and the question that matters most, whether XAI-enhanced AI demonstrably improves patient outcomes, has not been asked in a prospective trial. Three research priorities follow: developing standardized frameworks for evaluating explanation quality; conducting prospective trials comparing AI-with-XAI versus AI-alone on perioperative outcomes; and integrating causal inference methods to move from feature attribution towards actionable clinical recommendations.
